# Ischaemic conditioning and targeting reperfusion injury: a 30 year voyage of discovery

**DOI:** 10.1007/s00395-016-0588-8

**Published:** 2016-10-20

**Authors:** Derek J. Hausenloy, Jose A. Barrabes, Hans Erik Bøtker, Sean M. Davidson, Fabio Di Lisa, James Downey, Thomas Engstrom, Péter Ferdinandy, Hector A. Carbrera-Fuentes, Gerd Heusch, Borja Ibanez, Efstathios K. Iliodromitis, Javier Inserte, Robert Jennings, Neena Kalia, Rajesh Kharbanda, Sandrine Lecour, Michael Marber, Tetsuji Miura, Michel Ovize, Miguel A. Perez-Pinzon, Hans Michael Piper, Karin Przyklenk, Michael Rahbek Schmidt, Andrew Redington, Marisol Ruiz-Meana, Gemma Vilahur, Jakob Vinten-Johansen, Derek M. Yellon, David Garcia-Dorado

**Affiliations:** 1 The Hatter Cardiovascular Institute, University College London, London, UK; 2 The National Institute of Health Research University College London Hospitals Biomedical Research Centre, London, UK; 3 Cardiovascular and Metabolic Disorders Program, Duke-National University of Singapore, 8 College Road, Singapore, 169857 Singapore; 4 National Heart Research Institute Singapore, National Heart Centre Singapore, Singapore, Singapore; 5 Department of Cardiology, Vall d’Hebron University Hospital and Research Institute, Universitat Autònoma, Barcelona, Spain; 6 Department of Cardiology, Aarhus University Hospital Skejby, 8200 Aarhus N, Denmark; 7 Department of Biomedical Sciences and CNR Institute of Neurosciences, University of Padova, Padua, Italy; 8 Department of Physiology and Cell Biology, College of Medicine, University of South Alabama, Mobile, AL USA; 9 Department of Cardiology, Rigshospitalet, Copenhagen University Hospital, Copenhagen, Denmark; 10 Department of Pharmacology and Pharmacotherapy, Semmelweis University, Budapest, Hungary; 11 Pharmahungary Group, Szeged, Hungary; 12 Institute for Biochemistry, Medical Faculty Justus-Liebig-University, Giessen, Germany; 13 Department of Microbiology, Kazan Federal University, Kazan, Russian Federation; 14 Institute for Pathophysiology, West-German Heart and Vascular Center, University of Essen Medical School, Essen, Germany; 15 Centro Nacional de Investigaciones Cardiovasculares Carlos III (CNIC), Madrid, Spain; 16 IIS-Fundación Jiménez Díaz Hospital, Madrid, Spain; 17 2nd University Department of Cardiology, National and Kapodistrian University of Athens, Athens, Greece; 18 Duke University, Durham, NC USA; 19 Institute of Cardiovascular Sciences, University of Birmingham, Birmingham, UK; 20 Oxford Heart Centre, The John Radcliffe Hospital, Oxford University Hospitals, Oxford, UK; 21 Department of Medicine, Hatter Institute for Cardiovascular Research in Africa and South African Medical Research Council Inter-University Cape Heart Group, Faculty of Health Sciences, University of Cape Town, Chris Barnard Building, Anzio Road, Observatory, Cape Town, Western Cape 7925 South Africa; 22 King’s College London BHF Centre, The Rayne Institute, St. Thomas’ Hospital, London, UK; 23 Department of Cardiovascular, Renal, and Metabolic Medicine, Sapporo Medical University School of Medicine, Sapporo, Japan; 24 Explorations Fonctionnelles Cardiovasculaires, Hôpital Louis Pradel, Lyon, France; 25 UMR 1060 (CarMeN), Université Claude Bernard, Lyon 1, France; 26 Cerebral Vascular Disease Research Laboratories, University of Miami Miller School of Medicine, Miami, FL 33136 USA; 27 Neuroscience Program, University of Miami Miller School of Medicine, Miami, FL 33136 USA; 28 Department of Neurology, University of Miami Miller School of Medicine, Miami, FL 33136 USA; 29 Carl von Ossietzky Universität Oldenburg, Ökologiezentrum, Raum 2-116, Uhlhornsweg 99 b, 26129 Oldenburg, Germany; 30 Department of Physiology and Emergency Medicine, Cardiovascular Research Institute, Wayne State University, Detroit, MI USA; 31 Division of Cardiology, Department of Pediatrics, Heart Institute, Cincinnati College of Medicine, Cincinnati Children’s Hospital Medical Center, Cincinnati, OH USA; 32 Cardiovascular Research Center, CSIC-ICCC, IIB-Hospital Sant Pau, c/Sant Antoni Maria Claret 167, 08025 Barcelona, Spain; 33 Division of Cardiothoracic Surgery, Department of Surgery, Emory University, Atlanta, USA

**Keywords:** Ischaemic conditioning, Myocardial reperfusion injury, Cardioprotection, RISK and SAFE pathway, Mitochondria

## Abstract

To commemorate the auspicious occasion of the 30th anniversary of IPC, leading pioneers in the field of cardioprotection gathered in Barcelona in May 2016 to review and discuss the history of IPC, its evolution to IPost and RIC, myocardial reperfusion injury as a therapeutic target, and future targets and strategies for cardioprotection. This article provides an overview of the major topics discussed at this special meeting and underscores the huge importance and impact, the discovery of IPC has made in the field of cardiovascular research.

## Introduction

The year 2016 marks the 30th anniversary since Murry, Jennings and Reimer first discovered the phenomenon of ischaemic preconditioning (IPC) [180]. The seminal discovery in 1986, that brief episodes of ischaemia and reperfusion could dramatically reduce myocardial infarct (MI) size, gave rise to the field of cardioprotection, and has resulted in over 10,000 publications in the research literature. Over the last 30 years enormous efforts have been made to understand the mechanisms underlying IPC and have provided huge insights into the mechanisms of cardiomyocyte death during acute ischaemia/reperfusion injury (IRI), and the complex signalling pathways underlying cytoprotection within the cardiomyocyte and beyond. In addition, the last 30 years have witnessed enormous efforts to translate this endogenous cardioprotective strategy into the clinical setting for patient benefit. In this regard, the evolution of IPC to an intervention which could be applied at the time of reperfusion [ischaemic postconditioning (IPost)] [276] and to a remote organ or tissue [remote ischaemic conditioning (RIC)] [200] has facilitated the translation of IPC into the clinical setting.

**Fig. 1:**
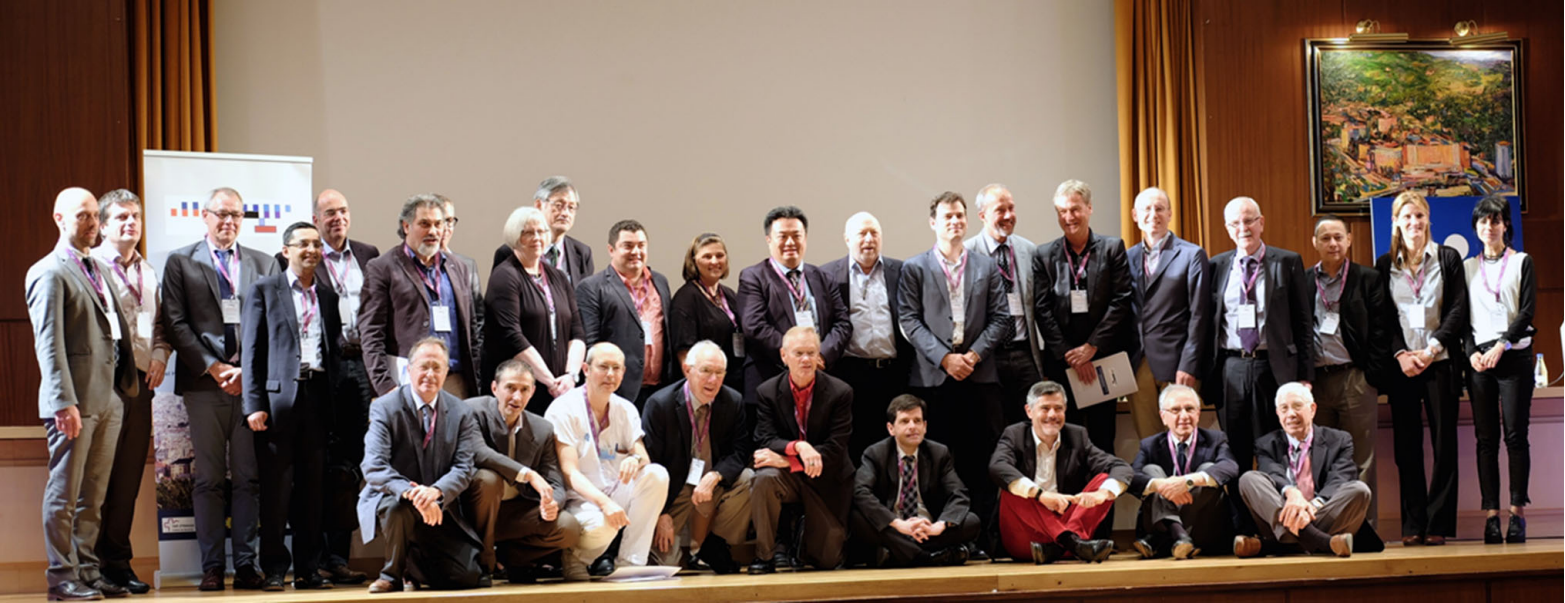
Faculty photo at the 30 year anniversary celebration of IPC in Barcelona May 2016: “Ischaemic conditioning and targeting reperfusion injury: a 30 year voyage of discovery”. *Back row*, *left* to *right* Michael Rahbek Schmidt, Peter Ferdinandy, Hans Erik Bøtker, Rajesh Kharbanda, Michael Marber, Pasquale Pagliaro, Thomas Engstrom, Karin Przyklenk, Tetsuji Miura, Hector A. Carbrera-Fuentes, Sandine Lecour, Derek Hausenloy, Derek Yellon, Borja Ibanez, Rainer Schulz, Gerd Heusch, Hans Michael Piper, Efstathios Iliodromitis, Miguel A Perez-Pinzon, Gemma Vilahur, Marisol Ruiz-Meana. *Front row, left* to *right* David Garcia-Dorado, Javier Inserte, Jose Barrabes, Robert Jennings, Jakob Vinten-Johansen, Andrew Redington, Michel Ovize, Fabio Di Lisa, James Downey

To commemorate the auspicious occasion of the 30th anniversary of IPC, leading pioneers in the field of cardioprotection gathered in Barcelona in May 2016 to review and discuss the history of IPC (Fig. 1), its evolution to IPost and RIC, myocardial reperfusion injury as a therapeutic target, and future targets and strategies for cardioprotection. This article provides an overview of the major topics discussed at this special meeting and underscores the huge importance and impact, the discovery of IPC has made in the field of cardiovascular research.

## Ischaemic preconditioning

In IPC, several minutes of acute coronary occlusion followed by reperfusion delay the onset of MI from a subsequent period of prolonged lethal ischaemia and reperfusion. The description of IPC 30 years ago in 1986 by Murry et al. [180] was a landmark discovery. It proved once and for all that the final size of a MI was not only a function of the area-at-risk (AAR), ischaemic time and collateral flow, but could indeed be reduced, as had been originally proposed by Braunwald and colleagues years before [165]. The Jennings laboratory was pursuing the observation that a brief ischaemic episode slowed the rate of ATP consumption when the heart was subjected to subsequent episodes of ischaemia. Since virtually no ATP is present in dead cardiomyocytes, they hypothesised that delaying ATP depletion would attenuate the development of cardiomyocyte death [181].

Considering the huge number of papers eventually published on IPC since 1986, it is amazing that it took 4 years before the first confirmatory paper by another laboratory appeared on the subject [149]. However, after that virtually everyone who tried to replicate IPC was able to observe protection that lasted for several hours [258]. In 1991, Liu et al. [153] showed that the preconditioned state resulted from protective signal transduction. Infusing adenosine or an adenosine A_1_ receptor-selective agonist into the coronary arteries for 5 min prior to occluding a coronary branch put the heart into a protected state identical to IPC. Conversely, an adenosine receptor antagonist completely blocked the IPC protection but had no effect on a non-IPC heart. A_1_ receptors are G_i_-coupled and act to slow the heart rate as opposed to the G_s_-coupled adenosine A_2_ receptors which act to dilate the coronary arteries. In fact it was shown that many of the G_i_-coupled receptors in the heart can mimic IPC [40]. A brief coronary occlusion has been found to release ligands for only four of these receptors: adenosine, bradykinin, opioid, and sphingosine. These four receptors act in an additive fashion. Blocking a single receptor subtype only raises the ischaemic threshold for protection rather than abolishing the IPC response. Subsequent studies quickly showed that protein kinase C [155] and ATP-sensitive potassium channels (K_ATP_) [5], which later turned out to be in the mitochondria [154] and could be stimulated by diazoxide (pharmacological preconditioning), were also in the IPC signalling pathway.

**Fig. 2:**
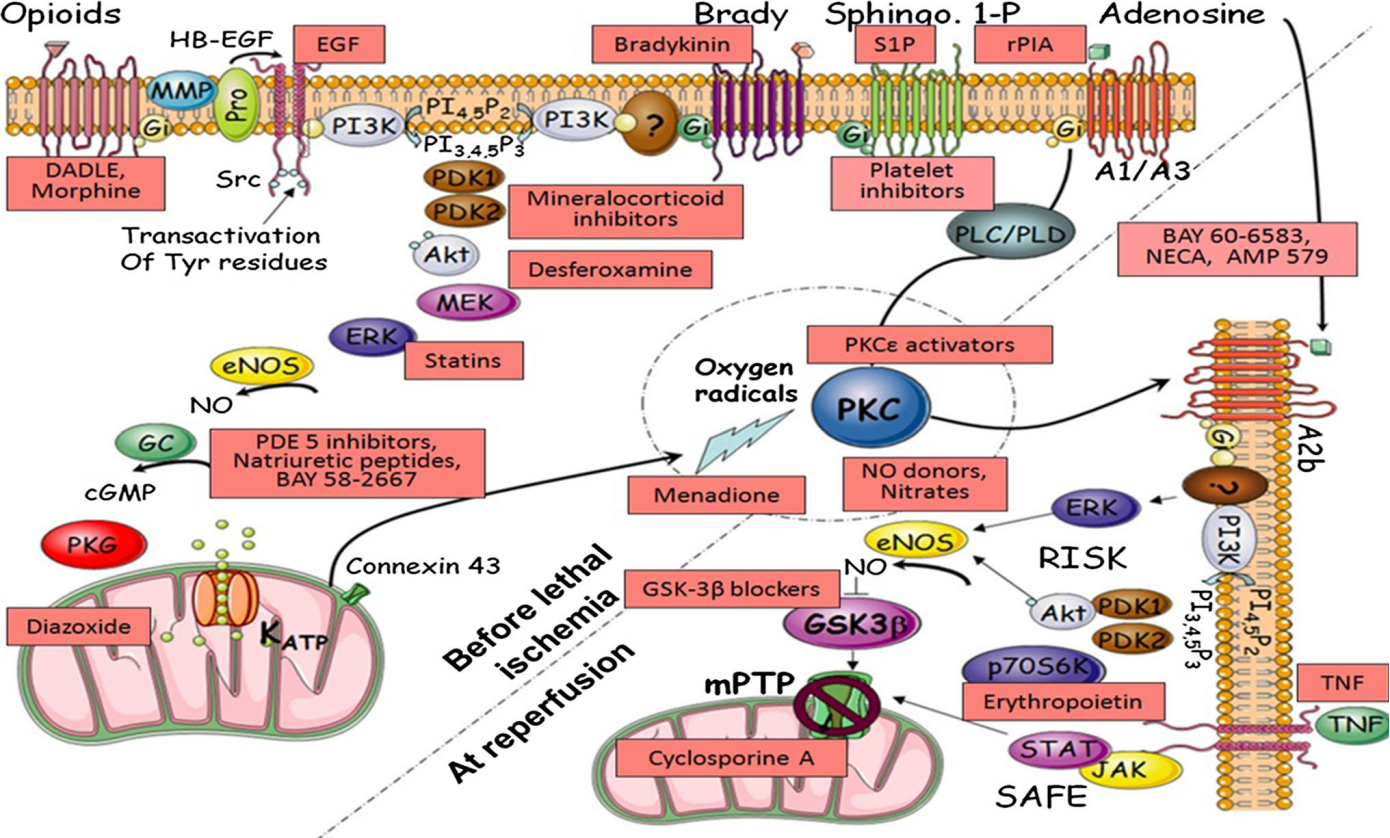
A proposed map of some of the major signalling pathways involved in ischaemic pre- and postconditioning. The *pink coloured boxes* indicate pharmacological interventions that have been reported to reduce MI size when administered just prior to reperfusion. They are positioned near their proposed site of action. Figure modified from that appearing in [38]

The overall signalling pathway is still not completely understood but extensive research in many laboratories has revealed much of it (Fig. 2) [29, 74, 101, 273]. In 2002, Yellon’s group [86, 94, 218] proposed the Reperfusion Injury Salvage Kinase or RISK Pathway to describe a group of pro-survival kinases that must be activated at the time of reperfusion for IPC to protect against MI. Since protection could be aborted by blocking the RISK pathway at reperfusion, IPC must, therefore, protect against a reperfusion injury. They also went on to demonstrate the importance of this pathway in all forms of the conditioning process, i.e. pre-, post-, remote and pharmacological conditioning [90]. It now appears that much of the cell death in the heart is due to the formation of permeability transition pores (PTPs) in the mitochondrial membranes in the first minutes of reperfusion, and IPC through the RISK signalling protects by suppressing these PTPs [97, 103]. Lecour et al. [146] subsequently identified the Salvage Activating Factor Enhancement (SAFE) pathway which is activated in parallel to the RISK pathway and appears to play a more important role in larger mammals [78, 108, 227, 229].

The key to understanding IPC is to appreciate why the brief period of reperfusion after the preconditioning ischaemia is so important. The G_i_ receptor activation leads to opening of mitochondrial K_ATP_ channels during ischaemia and potassium entry into the mitochondria. When oxygen is reintroduced during the reperfusion phase of the IPC protocol, elevated mitochondrial potassium stimulates the mitochondria to produce reactive oxygen species (ROS). These ROS cause redox signalling which ultimately results in PKC activation and completion of the IPC signalling pathway [52]. In a non-conditioned heart this pathway is blocked at the redox signalling step during the prolonged ischaemic period as potassium has entered the mitochondria but there is no oxygen available. If the heart is reperfused after a prolonged ischaemic period PTPs will always open before redox signalling can activate the downstream pathway to inhibit them [37]. A large series of recent studies have demonstrated that connexin 43, the protein forming gap junction channels between cardiomyocytes, is also located at the inner mitochondrial membrane [15], where it can form hemi-channels that allow the passage of potassium [175], and that its absence at that location prevents ROS generation during the IPC stimulus [98], and abolishes cardioprotection [205]. There is recent evidence that IPC can protect mitochondria against respiratory inhibition induced by prolonged IRI independently of cytosolic signalling [215].

Although IPC is clearly protective, the need for its application before ischaemia makes it impractical for treating acute myocardial infarction (AMI). However, if the protective pathway can be rapidly activated by a drug administered at reperfusion (known as “pharmacological postconditioning”) then this can “win the race” and protect the heart against reperfusion injury. Because much of conditioning’s signal transduction pathway is now known [101], it has been possible to identify agents effective at inducing pharmacological postconditioning. Figure 2 shows a tentative map of the signalling pathways underlying IPC, and some known interventions that, at least in animal models, reportedly put the heart into a conditioned state.

## Second window of protection

The second window of protection (SWOP) describes the increased resistance to myocardial injury that re-appears 12–24 h after the short durations of ischaemia/reperfusion that trigger classic or early preconditioning. This phenomenon was first described in 1993 by independent research groups based in London [164] and Osaka [140].

Yellon’s group in London had been interested in the cardiac protection that followed whole body heart stress—this was known to be associated with the induction of stress proteins and catalase within the myocardium [44]. However, whole body heat stress was associated with a multitude of changes both within and outside the heart. Using an experimental method that involved a support animal providing oxygenated blood to perfuse a donor animal’s isolated heart, evidence had been obtained that whilst whole body heat stress protected the heart, it also triggered extra-cardiac adaptations that aggravated myocardial injury [252]. This observation provided the impetus to find a way of spatially restricting the stress response to the heart, thereby avoiding the deleterious systemic adaptations associated with whole body heart stress. In 1991, Knowlton had observed cardiac stress protein induction beginning 6–8 h after short episodes of myocardial ischaemia [136]. Based on this observation, it was reasoned that sublethal myocardial ischaemia would induce stress proteins without causing the detrimental systemic response associated with whole body heat stress. It was on this basis that Marber et al. designed and performed the experiments that laid the foundation for the SWOP [164].

Following the original observations by Marber [164] and Kuzuya [139], there followed a number of basic science studies that indicated the SWOP had a duration of 72–96 h, and whilst the magnitude of protection may be less robust than that of the first window of protection, there were likely to be important clinical correlates [272].

Studies in patients have broadly fallen into two groups; observational studies, where symptoms and circumstance are related to outcome after spontaneous plaque rupture (type I MI) and interventional studies using controlled, iatrogenic myocardial ischaemia. In the observational studies, it was found that patients who experience repeated episodes of pre-infarction angina more than 24 h before the index event, may develop smaller final MI size than those without pre-infarction angina [102, 184]. Interpretation of these studies is complicated by the variation of each ischaemic episode in terms of its duration, intensity and exact timing before spontaneous coronary artery occlusion. Furthermore, most patients have co-morbidities and medications that have been shown to both facilitate and prevent the manifestation of protection. These uncontrollable variables may explain the discrepancies that appear in the literature regarding the benefit of pre-infarction angina. Consequently, the benefit of the SWOP is more easily demonstrated in interventional studies that use exercise treadmill tests, isotope scintigraphy or coronary angioplasty to cause and document myocardial ischaemia [14, 121, 143, 190].

In conclusion, the SWOP is clinically apparent under pre-specified controlled conditions but is impossible to identify with certainty in observational retrospective studies relevant to everyday clinical practice. Furthermore, the systematic changes in primary percutaneous coronary intervention (PPCI) services ensure early intervention with the surety of reperfusion further secured by advances in antiplatelet drugs and interventional devices [262]. On this background it is very difficult to demonstrate sufficient room for novel cardiac protection [28, 76, 99, 110, 262]. Furthermore, any benefit of SWOP may be subsumed by ischaemic pre- and/or post-conditioning. Nonetheless, one aspect of SWOP which may yet prove important is its potential to reveal novel protective proteins that may form the basis of future therapies.

## Remote ischaemic conditioning

Remote ischaemic conditioning is the intriguing phenomenon, first reported by Przyklenk, Whittaker and colleagues [200, 260], that brief periods of ischaemia applied in a distant tissue can render the heart resistant to IRI and reduce MI size. Although first viewed as a specious finding [201], the concept of RIC-induced cardioprotection has, during the past two decades, been corroborated in multiple, diverse models (reviewed in [29, 93, 104, 193, 226]). A recent meta-analysis of experimental studies in RIC found that RIPC reduced MI size as a percentage of AAR by 22.8 %, when compared to untreated controls, and RIPerC/RIPostC reduced MI size by 22.2 % [26]. Moreover, two priorities have emerged: (1) identification of the mechanisms responsible for the infarct-sparing effect of RIC; and (2) translation of RIC to patient cohorts.

The molecular mechanisms contributing to RIC are, without question, complex and remain incompletely resolved [30–32, 93, 101, 104, 193, 226]. In brief, the current hypothesis is that RIC induces a neuro-humoral response which, in turn, induces a cascade of downstream effects. Evidence for a neural component of RIC comes from early observations that pretreatment of animals with the ganglion blocker hexamethonium abolishes the cardioprotective effect of transient mesenteric ischaemia [68], and subsequent studies showing that transection of the ipsilateral femoral nerve [152, 235], or bilateral cervical vagotomy [50], abolishes cardioprotection by RIC induced by limb ischaemia. Conversely, direct stimulation of the femoral nerve [204] or sensory nerves [169] within the limb have been shown to induce cardioprotection. However, there are also controversial data on neuronal involvement in RIC, as hexamethonium [257] or nerve transection [203] did not abrogate cardioprotection. The consequence of any neural stimulus, whether local to the limb [221] or of the cardiac ganglia [194], is the release of dialysable cardioprotective substances into the blood [129]. These include the chemokine SDF-1α [47], Ribonuclease-1 [30], leukotrienes [224], and microRNA 144 [150]. The exact mechanism by which any of these putative effectors are released, and their relative importance remains to be fully understood. Yet, the ultimate effect at the cardiomyocyte level is to induce a protective kinase response [151, 227], and modification of PTP opening [247], similar to that observed with local preconditioning and postconditioning. Unlike local preconditioning and postconditioning, RIC appears to have additional pleiotropic effects that modify pathways involved in the acute and chronic responses to IRI and may contribute to its benefits, including improved vascular endothelial function [158], decreased platelet aggregation [10, 191], and a significant anti-inflammatory effect manifest early by decreased neutrophil adhesion [220], and later by reduced inflammatory cell infiltration, reduced local inflammation [30] and reduced remodelling in the weeks after experimental MI [256].

In the clinical setting, remote ischaemic preconditioning (RIPC) has been administered prior to IRI as three or four cycles of 5 min ischaemia followed by 5 min reperfusion of the upper, or less frequently, lower limb in cardiac and vascular surgery, and elective and emergency angioplasty. The majority of studies in coronary artery bypass graft (CABG) surgery patients have shown reduction of post-operative cardiac biomarker release [2, 33, 35, 84, 138, 239, 240, 250] while others did not [69, 131, 159, 167, 202, 275]. One randomized study of 329 CABG patients demonstrated simultaneous reduction of troponin I release and reduction of all-cause mortality up to 4 years following the operation [91]. In contrast, a randomised study of 1280 patients undergoing off-pump coronary artery bypass graft surgery showed no effect of RIPC before and after the surgery on a comprehensive composite endpoint [113]. Two more recent studies, the ERRICA and the RIPHeart studies, also failed to demonstrate any beneficial effect on major adverse cardiac and cerebral events (MACCE) after 12 months and event free survival after 3 months, respectively [77, 171]. Studies that failed to demonstrate a beneficial effect of RIPC used propofol as an anaesthetic regimen. Similar experiences have been obtained in major non-cardiac surgery [3, 254]. A specific effect of propofol that interacts with neuronal transfer of the protective RIPC signal may interfere with the inherent cardioprotective effect of propofol and further protection by RC [137, 138].

Invasive coronary procedures circumvent any influence from anaesthetics. In this setting, RIPC attenuated the release of ischaemic markers in the majority of studies including patients undergoing elective percutaneous coronary intervention (PCI) [114, 162, 198, 265, 277], and translated into a prognostic benefit in terms of MACCE at follow-up period of up to 6 years [49]. Whilst RIPC can be used in predictable ischaemia, another temporal variant is necessary in unpredictable ischaemia such as ST-segment elevation myocardial infarction (STEMI). Remote ischaemic perconditioning (RIPerC) [217], in which the RIC intervention is applied during evolving MI prior to PPCI, has consistently yielded cardioprotection in proof-of-concept studies using a variety of outcome measures including myocardial salvage, ST-segment resolution and biomarker release (Table 1) [25, 42, 54, 179, 199, 205, 259, 271]. The reduction of MI size translated into a reduction of MACCE [230] and was cost-effective [231] over a 4-year period following the index infarct. This study included 333 patients and was not powered for clinical outcomes. The ongoing CONDI-2/ERIC-PPCI study including 4300 patients will determine the clinical benefit of RIPerC as an adjunct to PPCI in patients with STEMI [79].

**Table 1:** Proof-of-concept studies of remote ischaemic conditioning in STEMI

Study	No of patients (control/RIC)	RIC regimen	Endpoint	Outcome
Bøtker et al. [25]	69/73	Upper limb 4 cycles I/R (5/5 min)	Salvage index (SPECT)	20 % increase in salvage index
Munk et al. [179]	110/108	Upper limb 4 cycles I/R (5/5 min)	LVEF at 30 days	5 % increase in LVEF in anterior infarcts
Rentoukas et al. [205]	30/33	Upper limb 3 cycles I/R (5/5 min)	ST-segment resolution	20 % increase in proportion of patients achieving full ST-segment resolution
Crimi et al. [42]	50/50	Lower limb 3 cycles I/R (5/5 min)	CK-MB (AUC 72 h after PCI)	20 % reduction of CK-MB release
Prunier et al. [199]	17/18	Upper limb 4 cycles I/R (5/5 min)	CK-MB (AUC 72 h after PCI)	31 % reduction of CK-MB release
Yellon et al. [271]	260/260	Upper limb 4 cycles I/R (5/5 min)	TnT (AUC 24 h after PCI)	17 % reduction of TnT release
White et al. [259]	40/43	Upper limb 4 cycles I/R (5/5 min)	Cardiac MRI	27 % reduction of MI size
Eitel et al. [54]	232/232/232	Upper limb 3 cycles I/R (5/5 min) + local IPost)	Salvage index (cardiac MRI)	23 % increase in salvage index

*AUC* area under curve, *CK-MB* creatine kinase-myocardial band, *LVEF* left ventricular ejection fraction, *MRI* magnetic resonance imaging, *PCI* percutaneous coronary intervention, *SPECT* single photon emission computerised tomography, *Tn* troponin

## Ischaemic postconditioning

Emergence of the concept of ischaemic postconditioning (IPost) was based on four points: (a) myocardial reperfusion injury was not a laboratory curiosity but an pathophysiological entity that exacerbated tissue injury (whether de novo or extending pre-existing injury) after onset of reflow; (b) lethal myocardial reperfusion injury was initiated quickly after the onset of reperfusion; (c) tissue destined to die in the path of the reperfusion injury “wave front” after onset of reflow could be salvaged; (d) reperfusion injury pathology could be avoided or prevented by altering how the ischaemic tissue was reperfused. The latter point was expanded to include modifying the conditions and composition of the reperfusate, including the inclusion of drugs during early reperfusion. Despite considerable controversy over the very existence and clinical importance of myocardial reperfusion injury, there is now compelling evidence that reperfusion contributes to the extent of transient as well as permanent (lethal) post-ischaemic injury to the myocardium [27, 65, 73, 251, 274], and that this injury was initiated within moments after onset of reflow [246]. Early reports of the protective effects of gradual or gentle reperfusion (modified conditions of reperfusion) in the early moments of reperfusion [22, 115, 216] did not capture the attention of the scientific or clinical communities. Although initial trials on IPost were performed in 1992, results were negative due to (a) excessively long durations of the reperfusion-re-occlusion cycles (5 min emulating preconditioning cycles), and (b) a single cycle rather than multiple cycles; studies resumed eight years later using shorter cycle durations in a large animal model which successfully reduced MI size, coronary artery endothelial dysfunction, oedema in the AAR, and apoptosis [276].

Studies confirming and extending the original results were published quickly by independent laboratories [63, 245, 270] as well as by Vinten-Johansen’s laboratory [71, 135]. Kin et al. [135] showed that the cardioprotective effects of IPost were not observed if the manoeuver was delayed by 60 s, confirming that a IPost window opened in the first few minutes of reperfusion which was critical to protection. This IPost window was confirmed by Yang et al. [270] and implied that reperfusion injury interventions should be implemented immediately at or before the onset of reperfusion. There is scant evidence that delayed postconditioning is effective in reducing post-ischaemic injury [209].

IPost has been shown to reduce abnormal alterations in a multitude of end points associated with post-ischaemic injury. These include reduction of (1) MI size and possibly the no-reflow area, (2) apoptosis, (3) interstitial and intracellular oedema, (4) early post-ischaemic arrhythmias, (5) the pro-inflammatory response to reperfusion, (6) explosive (injurious) ROS generation by multiple cell types, and (7) the incidence of heart failure. Whether IPost attenuates transient (stunning) or permanent contractile dysfunction globally or regionally is controversial. In addition, the cardioprotection of IPost may be lost in the presence of comorbidities (diabetes, hypertension, and hypercholesterolemia) or co-medications (such as P2Y12 inhibitors), in older individuals [19, 56, 225]. Although these data may imply limited efficacy in patients that present with isolated or the constellation of comorbidities in the metabolic syndrome, it must be said that efficacy has been shown in patients that present for PCI with these demographics [192].

The mechanisms by which these physiological responses to reperfusion are attenuated include (1) inhibiting PTP opening [4, 85, 103], (2) delaying rapid myocardial re-alkalinisation that, in part, contributes to PTP opening [39], (3) reducing intracellular and intra-mitochondrial calcium accumulation [117], (4) reduced oxidative damage of eNOS and preserved cGMP signalling [125], (5) attenuating endothelial dysfunction (expression of adhesion molecules [276], production of NOˑ and other vasoactive and cardioprotective autacoids such as adenosine) that otherwise trigger the vascular inflammatory response to reperfusion injury, and (6) reducing pro-inflammatory cell activation and expression of cytokines in blood that contribute to the inflammatory response to reperfusion injury [31, 32]. A year after its introduction, Tsang et al. [245] reported that IPost activated the reperfusion injury salvage kinase (RISK) pathway pro-survival kinases PI3K-Akt and downstream targets eNOS and p70S6K (see later section).

Unlike preconditioning whose clinical application is limited by the unpredictability of AMI, IPost immediately caught the attention of interventional cardiologists. In 2005, Staat et al. [234] reported that four episodes of 1-min inflation–deflation cycles of the angioplasty balloon performed immediately after coronary artery re-opening were able to significantly reduce MI size in STEMI patients. This was the first report demonstrating that reperfusion injury exists in man, is of pathophysiological importance, and can be attenuated by a timely intervention. Most [133, 238, 241], but not all [132], clinical studies in patients with these conditions undergoing PCI or cardiac surgery have shown positive outcomes with IPost (reviewed in Heusch [99, 101, 110]). Reasons for such discrepancy are unclear but might include a different use of thrombus aspiration, direct stenting, in-stent balloon inflation–deflation for inducing IPost, as well as the confounding role of new adjunct therapies like P2Y12 inhibitors. The recent phase 3 DANAMI-3 IPOST study (NCT01435408) [111] reported that 4 cycles of 30 s IPost failed to improve clinical outcomes in STEMI patients, but this study used a sub-optimal IPost algorithm and was probably underpowered. Additional studies are awaited to clarify whether or not MI size reduction observed in phase 2 IPost trials can actually provide any clinical benefit to STEMI patients.

Whether IPost is cardioprotective in the presence of comorbidities such as diabetes or hypercholesterolaemia, or in the presence of co-medications, or wanes with age [19] is still controversial [56, 192]. In addition, whether the efficacy of IPost is masked when other forms of cardioprotection are used, such as IPC, P2Y12 inhibitors, or hypothermia and cardioplegia in cardiac surgery, is still unresolved.

Studies should continue to unravel the numerous and interacting mechanisms involved in IPost, and how they relate to other types of conditioning (preconditioning, perconditioning). In addition to clarifying the mechanisms of IPost, these studies may lead to the development of broad spectrum drugs with multiple therapeutic targets emulating IPost’s broad spectrum therapeutic profile. Potential loss of cardioprotection in comorbid circumstances should be further investigated in large animal models with genetic predispositions to the comorbidity spectrum, such as the Ossabaw pig with genetic metabolic syndrome. Similar studies need to define whether efficacy of IPost is lost with advancing age. Studies in large animal models should re-examine the “ischaemic wavefront” to separate the temporal progression of myocardial injury after ischaemia only (without reperfusion) and after ischaemia plus reperfusion to redefine the extent of injury in patients arriving at the catheter laboratory with unresolved occlusions. More clinical studies need to be performed that embrace the design features of randomisation and adequate statistical power that avoids Type II errors, and allows stratification of patients into various subgroups to differentiate responders from non-responders. A combination of different protective interventions, including remote, per- and postconditioning as well as cocktails of drugs may be tested in the future.

## Myocardial reperfusion injury

Reperfusion Injury has many facets. Apart from reversible forms of reperfusion injury, including reperfusion arrhythmias and stunning, there is also lethal reperfusion injury, or cell death, occurring at the time of reperfusion, and thus preventable by treatments applied at the time of restoration of blood flow [196]. There is extremely solid evidence of the existence of lethal reperfusion injury in experimental MI models. There is also solid evidence of the occurrence of reperfusion injury in patients with STEMI, although several interventions to reduce lethal reperfusion injury in patients have failed or provided inconsistent results in this clinical setting [76, 106, 110]. The reasons for these failures are more likely dependent on the particular treatments applied or on associated circumstances (age, comorbidities, treatment received) than to inter-species differences. Over the past 30 years, the importance and mechanisms of cardiomyocyte cell death in myocardial reperfusion injury have been elucidated in part. Altered Ca^2+^ handling and PTP opening have been identified to be complementary pathways of reperfusion-induced cell death, but important questions remain unsolved [118].

Cardiomyocyte death is the main cause of heart failure, arrhythmias and death in patients with STEMI, and depends largely on phenomena occurring within cardiomyocytes themselves, as shown by the fact that it can be recapitulated in isolated cardiomyocytes submitted to transient ischaemia [118], but other cells can contribute, in particular, platelets [8, 9, 174]. Endothelial cells, in which metabolism is largely independent of mitochondrial respiration [163] are more tolerant of ischaemia than cardiomyocytes.

A substantial component of reperfusion-induced cell death occurs during the initial minutes of reperfusion [91]. Apoptosis plays little, if any, role in reperfusion-induced cardiomyocyte cell death [168] and selective lack of expression of executioner caspases 3 or 7, does not modify MI size or post-MI remodeling in mice [122]. Severe ischaemia stops mitochondrial respiration, progressively dissipates mitochondrial membrane potential, and ATP concentration reaches very low levels and triggers rigor contracture [188]. ATP hydrolysis secondary to reversal of respiratory Complex V (ATP synthase) plays an important role [59]. The inactivity of the Na^+^ ATPase pump leads to Na^+^ and Ca^2+^ overload through reverse Na^+^/Ca^2+^ exchange [196]. Anaerobic metabolism in combination with reduced catabolite washout causes intracellular acidosis, reaching pH 6.4 within a few minutes. Reperfusion results in the rapid restoration of energy availability [59] and intracellular pH [126], and generation of large amounts of reactive oxygen species (ROS) and additional Ca^2+^ influx [64].

Altered Ca^2+^ handling is a key factor in reperfusion injury-cardiomyocyte cell death. Na^+^ concentration may increase in reperfused cardiomyocytes due to Na^+^ influx associated with pH normalization and passage of Na^+^ from adjacent cells via gap junctions favoured by impaired Na^+^ pump function [213], and Na^+^ influx favours Ca^2+^ influx through the Na^+^/Ca^2+^ exchanger [164]. Increased Ca^2+^ and pH normalisation causes calpain activation [124] resulting in damage of the subsarcolemmal cytoskeleton leading to Na^+^ pump dysfunction. Restoration of ATP availability during initial reperfusion leads to Ca^2+^ uptake into the sarcoplasmic reticulum (SR) followed, when Ca^2+^ capacity is exceeded, by Ca^2+^ release through the Ryanodine receptor channel (RyR2) resulting in oscillations of Ca^2+^ concentration that propagates across the cell favouring arrhythmias, hypercontracture and mitochondrial Ca^2+^ overload [210]. Hypercontracture can cause cell death, and transient contractile inhibition during the initial minutes of reperfusion prevents cardiomyocyte death in isolated cardiomyocytes, isolated hearts and intact large animals [66, 222]. Reperfusion-generated ROS cause nitric oxide synthase (NOS) oxidation and reduced NO-cGMP-PKG signaling. PKG modulates phospholamban (PLB) phosphorylation, SR Ca^2+^ uptake, and Ca^2+^ oscillations, and treatments normalizing PKG signaling in reperfused myocardium limit MI size in a number of pre-clinical and clinical studies [123].

Resumption of respiration is associated with increased ROS generation, due in part to re-oxidation of succinate accumulated during ischaemia and reverse electron transport between complex II and complex I of the respiratory chain [36] and to restoration of mitochondrial potential favoring mitochondrial Ca^2+^ uptake through the Ca^2+^ uniporter and Ca^2+^ overload [64]. Studies in mitochondrial preparations and cells show that mitochondrial Ca^2+^ and ROS may trigger an abrupt increase in the permeability of the inner mitochondrial membrane resulting in release of molecules from the mitochondrial matrix into the cytosol, mitochondrial depolarisation and swelling [85, 185, 186]. Mitochondrial permeability transition is supposed to be due to the opening of the PTP, a proposed large conductance cannel in the inner mitochondrial whose molecular structure is not really clear [103], except for the involvement of cyclophilin D and more recently ATP synthase. PTP opening is inhibited by low pH and favored by ROS and low ATP concentration, conditions occurring during myocardial reperfusion [72].

Mitochondrial permeability transition and Ca^2+^ oscillations/hypercontracture are closely related cell death pathways. This is partly due to the tight physical connection between SR and mitochondria allowing preferential Ca^2+^ exchange between both organelles [212]. Ca^2+^ release from mitochondria secondary to PTP opening may cause hypercontracture in Ca^2+^ overloaded cardiomyocytes [211] while SR-driven Ca^2+^ oscillations may cause PTP opening [210]. The relative importance of these two pathways may depend on conditions such as the severity of the ischaemic insult [214].

Opening of the PTP has been well documented in mitochondrial preparations exposed to very high Ca^2+^ concentrations and isolated cardiomyocytes subjected to simulated IRI [72]. The most important evidence for the role of PTP in reperfusion injury is the reduced MI size associated with genetic ablation of cyclophilin D [6, 75, 81, 183]. Inhibition of cyclophilin D with cyclosporine-A (CsA) prevents PTP opening in mitochondrial preparations but reductions in MI size with this agent have not been consistent, particularly when applied exclusively at the time of reperfusion or in large animals [130, 228], and a positive proof-of-concept trial in patients with STEMI [195] was not confirmed in a larger phase III trial [43]. Furthermore, all other drugs aimed at PTP inhibition in STEMI patients have so far failed. Although disappointing, these results are more likely explained by ineffective PTP inhibition by CsA, rather than the PTP not being important in human reperfusion injury.

## Reperfusion signalling via the RISK and SAFE pathway

It is now well established that ischaemic conditioning protects the heart from acute IRI through the activation of signal transduction pathways recruited at the onset of reperfusion. These signalling cascades mediate the cardioprotective signal elicited by ischaemic conditioning from the sarcolemma to the mitochondria and include, amongst others, the reperfusion injury salvage kinase (RISK), and the survivor activating factor enhancement (SAFE) pathways (reviewed in [80, 92, 94, 145, 146]).

The RISK pathway refers to the pro-survival kinases, Akt and Erk1/2, the activation of which at the onset of reperfusion reduces MI size [92, 94]. It was first described by Yellon and colleagues in 2002 while studying the signalling mechanisms underlying the cardioprotective effect induced by the growth factor, urocortin [218]. In that study the administration of urocortin specifically at the time of myocardial reperfusion reduced MI size and increased the phosphorylation of myocardial Erk1/2, the effects of which were abrogated by the co-administration of the pharmacological MEK1/2-Erk1/2 inhibitor, PD98059, at the time of reperfusion [218]. A large number of experimental studies have linked the activation of the RISK pathway to the cardioprotection induced by a diverse variety of pharmacological agents including growth factors, cytokines, and other agents such as metformin and statins [92, 94]. The RISK pathway has also been shown to mediate the cardioprotection induced by IPC and IPost, suggesting that it may be a common pathway for cardioprotection [86, 87, 245]. Most of the experimental studies implicating the RISK pathway as a cardioprotective pathway have been performed in small rodent models of AMI, whereas recent studies suggest that the RISK pathway does not appear to mediate the cardioprotection induced by IPost [132, 229], gentle reperfusion [182] or RIC [1, 227] in large animal models, suggesting species differences in the reperfusion signalling pathways underlying ischaemic conditioning.

In 2005, Lecour and colleagues made the unexpected observation that the MI-limiting effects of TNF-α at the onset of reperfusion were mediated independently of the RISK pathway [147, 148]. They subsequently discovered, that TNF-α administered at the onset of myocardial reperfusion recruited an alternative signalling cascade, termed the SAFE pathway [142, 145, 146, 148], by binding to TNF receptor type 2 and activating Janus Kinase (JAK) and Signal transducer and activator of transcription 3 (STAT3) via mechanisms which are still unclear but may involve sphingosine kinase [61]. A number of experimental studies have demonstrated that pharmacological conditioning mimetics which limit myocardial reperfusion injury do so via the activation of the SAFE pathway, including high density lipoproteins [62], melatonin [144], glyceryltrinitrate, and cariporide [140]. In IPC studies, the activation of the SAFE pathway was demonstrated to occur at two time-points, following the IPC protocol and at the onset of reperfusion [148, 236]. The activation of the SAFE pathway by ischaemic conditioning has been confirmed both in small and large animals of AMI [227], whereas in humans, the STAT-5 isoform appears to be preferentially activated [109].

It has been demonstrated that there exists crosstalk between the Akt and Erk1/2 components of the RISK pathway such that the pharmacological inhibition of one kinase activated the other kinase to ensure maximal protection against myocardial reperfusion injury [83]. Interestingly, a crosstalk also exists between the RISK and the SAFE pathways [232]. Both signaling pathways converge on mitochondria where they appear to mediate their cardioprotective effect by inhibiting PTP opening [16, 46]. The mechanism for this is unclear for the RISK pathway, but for the SAFE pathway, STAT-3 has been shown to be present in mitochondria, where it modulates mitochondrial respiration and targets the PTP [16, 108]. TNF-α itself can also directly target mitochondrial function [141].

Whether targeting the RISK and SAFE pathway can benefit patients subjected to acute myocardial IRI has not been directly tested, although pharmacological agents such as atrial natriuretic peptide, erythropoietin and statins, which are known to activate components of these two signaling cascades, have been investigated in the clinical setting with mixed results [160, 161]. Further studies are required to test whether using combination therapy to simultaneous target the RISK, SAFE and other pathways is a more effective cardioprotective strategy than focusing on one single signaling cascade.

## Mitochondria as targets of cardioprotection

The apparent paradox of IPC, whereby a short period of ischaemia protects from an otherwise lethal ischaemic episode, is reflected and likely contributed to, by paradoxical actions of Ca^2+^ and ROS in mitochondria. Undoubtedly, a large and prolonged elevation in Ca^2+^ and ROS levels causes cell death mainly by favoring PTP opening [13]. However, antioxidants abolish IPC protection [189], that is mimicked by a mild elevation in ROS or Ca^2+^ levels [264, 266]. Several processes have been proposed to explain the paradoxical involvement of ROS and Ca^2+^ in both survival and death of cardiomyocytes. First, intra-mitochondrial Ca^2+^ is necessary to stimulate oxidative phosphorylation by activating key dehydrogenase steps, while sub-lethal levels of ROS activate signaling pathways promoting cell survival [128]. A mild ROS formation has been proposed also to explain the protection related to the opening of mitochondrial K_ATP_ channels downstream of PKC-ɛ activation and upstream of PTP inhibition [41].

Besides the elucidation of the molecular nature of PTP, K_ATP_ channels and other potential targets of IPC protection, a major challenge in the field is to determine the threshold separating physiological from pathological levels of ROS and Ca^2+^. This issue can be addressed by exploiting technological advances in Ca^2+^ and ROS imaging that have largely contributed to our understanding of IRI and IPC protection. Early studies by Michael Piper using the Ca^2+^-sensitive, fluorescent dye Fura-2 demonstrated that the recovery of ATP production in isolated cardiomyocytes with Ca^2+^ overload during reoxygenation leads to Ca^2+^ oscillations and hypercontracture [223]. It was proposed that Ca^2+^ microdomains in the vicinity of the SR could raise local [Ca^2+^] to levels sufficient to drive mitochondrial Ca^2+^ entry. Subsequent experiments using the mitochondria-targeted, Ca^2+^-sensitive photoprotein, aequorin, helped to validate the concept of these Ca^2+^ “hot spots”. Importantly, mitochondrial Ca^2+^ uptake contributes to the buffering of cytoplasmic Ca^2+^ peaks in cardiomyocytes [53]. However, during reperfusion massive cytosolic Ca^2+^ oscillations can lead to mitochondrial Ca^2+^ overload and PTP opening [64]. Oxidative stress during reperfusion can accentuate SR Ca^2+^ release [45]. These cytosolic and mitochondrial Ca^2+^ changes also occur in perfused hearts during IRI, as was initially demonstrated by imaging Ca^2+^ using fluorescent dyes and microscopy [248], and more recently with genetically encoded reporters and multiphoton microscopy [48]. IPC was shown to attenuate ischaemic SR Ca^2+^ overload in the isolated rabbit heart [34].

The elucidation of signalling pathways related to IPC-induced protection commenced with the seminal discovery of the involvement of the adenosine receptor in IPC [153]. Several of the described pathways converge on cytosolic Akt and/or ERK, which lead to the activation of mitochondrial PKC-ɛ [177]. Activated mitochondrial PKC-ɛ induces not only opening of the mK_ATP_ channel but also activation of Akt-GSK3β signalling in mitochondria, both of which contribute to inhibition of PTP opening [176]. Despite the redundancy of IPC-induced signal pathways in the cytosol and mitochondria, several diseases have been shown to significantly impair IPC-induced signalling in the myocardium. Interestingly, diabetes mellitus attenuates activation of Akt in response to upstream signals [116, 173, 244, 245] and also lowers the threshold for PTP opening by enhanced mitochondrial recruitment of non-phosphorylated GSK3β [172, 237] and increased ER stress [127]. Recently, progress has been made in our understanding of PTP and mitochondrial Ca^2+^ uniporter. However, the intra-mitochondrial localisation of protein kinases and phosphatases and their relationships with PTP, ROS and Ca^2+^ regulating machineries remain unclear and warrant further investigation.

Beyond its participation in signal transduction during IPC, mitochondria are effectors of cardioprotection. IPC attenuates IRI-induced mitochondrial respiratory failure and oxidative damage independently of PTP opening, even in the absence of cytosolic components [178]. Importantly, Cx43 translocates to and is predominantly present at subsarcolemmal mitochondria [20] and subsarcolemmal mitochondria but not interfibrillar mitochondria are the main targets of IRI damage and IPC protection [20, 206]. Indeed, mitochondria with genetic ablation of Cx43 are resistant to preconditioning [20, 206, 212]. Mitochondrial Cx43 regulates complex 1-mediated respiration [18, 175], ROS production [98] and K^+^ permeability [175], although its participation in IRI pathophysiology remains unclear in detail. The role of subsarcolemmal mitochondria in cardioprotection is necessarily linked to the function of interfibrillar mitochondria, as the latter are involved in cytosolic calcium buffering, energy demand–supply matching and antioxidant regeneration through privileged communication with SR [51, 212]. Indeed, partial disruption of mitochondria-SR interplay appears to aggravate IRI-induced cytosolic calcium, hypercontracture, PTP opening and cell death in aged mice [59, 60]. Variations in mitochondrial Cx43 contents might explain the resistance against IPC-mediated cardioprotection observed in aged animals [17, 19, 219].

## Pharmacological targeting of myocardial IRI

Elucidation of the signaling pathways underlying ischaemic conditioning cardioprotection has identified a large variety of therapeutic targets for pharmacological cardioprotection. In this section, we review some of the more recent pharmacological therapies which have been investigated in the clinical setting to target myocardial IRI in reperfused STEMI patients.

### GLP-1

Glucagon-like peptide-1 (GLP-1) is an incretin hormone that regulates plasma glucose, and within the latest 10 years GLP-1 analogues have been introduced for treatment of type-2 diabetes [89, 112]. In addition, receptors for GLP-1 have been found in the heart [7]. In experimental studies GLP-1 or its analogues protect against reperfusion injury-induced cell death [23, 24, 88, 89, 243]. These cardioprotective analogues include exendin-4, a peptide derived from the saliva of the Gila lizard showing a GLP-1-like potency and efficacy at GLP-1 receptors [95, 255]. Exendin-4 was found to be cardioprotective during reperfusion in isolated rat hearts [233], a finding that has been confirmed in several species, e.g. pigs [242].

Intravenous (IV) exenatide was found to increase myocardial salvage by 15 % if administered as a 6 h infusion initiated 10 min before reperfusion in STEMI patients [157]. When examining only patients with short ischaemic times (<132 min) MI size was reduced by 30 % [156]. This effect of exenatide was confirmed in an Asian population, since Woo et al. observed an almost 50 % reduction in MI size when administered subcutaneously [263]. A recent clinical study has failed to demonstrate a cardioprotective effect with exenatide in STEMI patients—it is not clear why this study was neutral, but it may have been related to the dose used [208]. To date no trials have sought to challenge the results from the proof-of-concept studies on a clinical end point.

### Cyclosporin-A

Opening of the PTP is a critical signalling hub in the cascade of myocardial reperfusion injury and preventing it from opening has been suggested to be an obvious pharmacological target [82, 85, 185, 186]. CsA is a compound that preserves PTP closure and in addition, it has been reported to affect remodelling following MI [170].

In a small proof-of-concept study Piot et al. demonstrated that CsA can reduce enzyme leakage by 40 % and MI size by 20 % when administered as an IV bolus prior to PPCI [96, 195]. Both infarcts located in RCA and LAD were included but only patients with TIMI 0 were eligible. The more recent CYCLE trial recruited 410 STEMI patients within 6 h of symptom onset (TIMI flow grade 0–1) and randomized them to CsA (2.5 mg/kg) or control [187]. The primary endpoint (ST-segment resolution at 60 min) and secondary endpoints (high-sensitivity cardiac troponin T (hs-cTnT) on day 4, left ventricular (LV) remodelling, and clinical events at 6-months follow-up) were not reduced by CsA [187]. Finally, in the definite hard endpoints-powered CIRCUS trial, 970 anterior STEMI patients (TIMI flow 0–1 in the LAD) were randomised to CsA or placebo. The trial failed to show any effect of IV CsA on a composite endpoint of death, hospitalization for heart failure and adverse LV remodelling [195]. The reasons for CsA to improve clinical outcomes in STEMI patients are not known and have been discussed in several recent articles [76, 100].

### Metoprolol

Early beta-blocker therapy in reperfused STEMI patients is controversial and had largely been investigated in the pre-reperfusion era. However, recently it has been shown that IV metoprolol administered prior to reperfusion in a porcine model reduced MI size [120]. This experimental work was followed by a clinical trial (METOCARD-CNIC trial) demonstrating that IV metoprolol administered in the ambulance prior to PPCI reduced MI size and improved clinical outcomes (as a secondary endpoint) in anterior STEMI patients presenting early (<6 h) [119, 197]. More importantly, in the METOCARD-CNIC trial, patients receiving pre-reperfusion IV metoprolol had not only CMR-evaluated smaller infarctions [166], and better long-term left ventricular ejection fraction (LVEF) [197], but also the incidence of LV severe systolic dysfunction was significantly reduced [197]. Very recently, the results of the EARLY BAMI trial have been reported. This trial recruited 600 STEMI patients (any location) presenting within 12 h from symptoms onset. Patients were randomized to IV metoprolol (10 mg) or placebo [207]. Primary endpoint was MI size assessed by CMR one month after infarction. The trial was neutral and MI size was not smaller in patients allocated to IV metoprolol. There were no signs of adverse effects in patients receiving IV metoprolol, and the incidence of ventricular fibrillation was significantly lower in metoprolol-treated patients. These data support the safety of this strategy in Killip I–II STEMI patients. There are important differences between the METOCARD-CNIC and EARLY BAMI trials. Dose and timing of IV metoprolol administration were different between trials. In contrast to the METOCARD-CNIC trial, in the EARLY BAMI study, patients received only one 5 mg dose at recruitment, and per protocol the second dose was given in the catheter laboratory immediately before PCI. In fact, the first dose of metoprolol did not have any effect on blood pressure or heart rate, suggesting an underdosing effect. In this regard, a recent subanalysis from the METOCARD-CNIC trial demonstrated that the longer the “onboard” metoprolol time at the time of reperfusion, the higher the infarct-reduction effect [67]. In fact, patients receiving IV metoprolol close to reperfusion had a very mild protective effect, while those with a longer time from metoprolol 15 mg bolus to reperfusion were those with the largest reduction in MI size and improvement in long-term LVEF. These differences in dose and timing of metoprolol administration might explain the different conclusions from both trials. Given the clear safety profile and the low cost of this therapy, it is worth to continue the clinical research and perform a definite large hard endpoint-powered trial. In the near future, the MOVE ON! Trial will be initiated and more than 1200 anterior STEMI patients will be recruited and randomized to IV metoprolol (15 mg immediately after diagnosis is made in the out of hospital setting) or placebo. The primary endpoint will be the composite of cardiovascular death, heart failure, ICD insertion, or severe LV dysfunction.

### P2Y_12_ inhibitors

State of the art anti-thrombotic therapy in STEMI patients includes the early administration of P2Y12 inhibitors. Ticagrelor, a potent P2Y12 inhibitor was associated with reduced mortality in ACS patients when compared to another P2Y12 inhibitor (clopidogrel) [253]. These benefits may not be fully explained by a pure antiplatelet effect. In this regard, ticagrelor has been shown, to increase the levels of extracellular adenosine [21], a mediator known to exert a wide range of benefits including vasodilation, inhibition of platelet aggregation and leukocyte adherence to the vessel wall. In line with this, cangrelor, a potent and fast acting IV P2Y12 inhibitor, has been shown to reduce MI size in mouse [12], rat [267], rabbit [268], and primates [269]. Interestingly, the protection conferred by cangrelor is dependent upon the presence of platelets with no evidence of protection ex vivo in crystalloid-perfused Langendorff heart [267, 268]. This protection is mediated through pathways typically recruited by ischaemic conditioning, suggesting that P2Y_12_ inhibition, via a blood component, leads to conditioning-like protection [267, 268]. Therefore, IV P2Y_12_ inhibition may thus have the dual advantage of optimising both platelet inhibition and offering cardioprotection.

### Combination reperfusion therapy—a novel therapeutic strategy

As can be seen above, most attempts to reduce MI size in STEMI patients have relied on using a single agent to target one single component of myocardial IRI. However, myocardial IRI is the result of several mechanisms and thus targeting on individual phenomena will unlikely reduce the MI size. The possibility of targeting several mechanisms simultaneously (either with one agent targeting different pathways or by several agents administered simultaneously) is attractive although not widely undertaken. A recent large animal study [1] showed that the combination of RIC with glucose-insulin-potassium and exenatide had an additive benefit in terms of MI size reduction. The COMBAT-MI trial (NCT02404376) will test the potential benefits of using RIC with exenatide on MI size reduction in STEMI patient.

## Problems in translation to the clinic and confounding factors

Although much effort has been taken to translate cardioprotection into clinical practice, so far translation has not been successful, as still no drugs are on the market and no therapeutic interventions are available for routine clinical practice that may protect the heart after IRI and thereby prevent the development of post-ischaemic heart failure [11, 28, 76, 99, 106, 107, 110, 118]. There are two major problems of clinical translation to overcome in the future: (1) target discovery and validation taking into consideration the known confounding factors of cardioprotection; and (2) better design of clinical development studies.

The putative mechanisms of cardioprotection explored in the past three decades have so far led to potential drug targets that were not robust enough for their pharmacological use as clinical trials targeting them largely showed no efficacy. Although it has been known from preclinical studies already in the mid-1990s that major cardiovascular co-morbidities and risk factors including aging, hyperlipidemia, diabetes (see for the first extensive review from 1998 on the effect of risk factors on cardioprotection [58], as well as some later specific reviews on aging [19], hyperlipidaemia [55], and diabetes [178, 261]) and also their medications (see for the first extensive review: [57] and its updated version: [56]) largely modify the response of the ischaemic heart to cardioprotective therapies, target discovery and validation were performed and still continue to be performed in young and healthy animals in the vast majority of studies. In the future, at least the major known co-morbidities and their major classes of pharmacological treatments should be used to validate the potential drug target before entering into clinical trials. The second reason may be a simplified and biased way of target selection so far. It is already known from transcriptomics data from the early 2000 years that IRI and cardioprotection trigger multifactorial mechanisms, moreover, co-morbidities and co-medications also significantly modify the cardiac gene expression profile (see for extensive reviews: [249]). Therefore, targeting a single pathway to protect all IHD patients is obviously not an approach that may lead to success. In the future, maybe a multi-omics approach including transcriptomics, proteomics, and metabolomics followed by systems biological network analysis may provide novel targets using this unbiased global approach (see for extensive reviews: [249]).

Although clinical trials of RIC may show some patient benefit in acute MI patients with multiple co-morbidities [104], two recent large clinical studies in patients undergoing cardiovascular surgery (ERICCA, RIPHeart) [77, 171] revealed no evidence for protection. It must be mentioned, however, that in both of these trials propofol was used for anesthesia, although propofol has been shown before to interfere with the efficacy of conditioning [105, 106]. Some larger clinical studies of IPost so far showed no acute cardioprotection [70, 134] nor long-term benefit up to 1 year follow up [71]. Nevertheless, the results of these clinical studies showed that both RIC and IPost have a favorable safety profile [110], so further studies are encouraged using better design and enough power to find out the importance of confounding factors of ischaemic conditioning in clinical reality.

As to the clinical development of cardioprotective drugs, the results so far have been disappointing. Targeting mitochondria by PTP inhibitors and other mitochondrial protective compounds (CIRCUS, CYCLE, Bendavia, Mitocare, EMBRACE STEMI studies) or replacing NO by inhaled NO (NOMI trial) or nitrite administration were ineffective in clinical trials (see for a recent review [76]). It should be noted that the molecular targets of these drugs were not validated properly before entering into clinical trials, i.e. targets were selected by the traditional biased way and no validations have been attempted in any of the animal models with the presence of the confounding factors. In the future, based on careful preclinical validation, phase 2 studies with careful patient selection based on the relevant confounding factors for the specific molecular target(s) may open new perspectives for successful translation of cardioprotection. Also, given the complexity of the cardioprotective signal transduction [101], combined treatment of several targets maybe needed.

## Conclusions

In this article we have provided an overview of the major topics discussed at this special meeting to celebrate 30 years of research in the field of IPC and cardioprotection. The huge research literature, which has arisen from the seminal discovery of IPC, has provided important insights into the mechanisms and elucidation of the signalling pathways underlying cytoprotection in the heart and other organs. The evolution of IPC to both IPost and RIC has helped facilitate the translation of this endogenous cardioprotective strategy from the laboratory to the clinical setting. We hope this article provides a worthy account of the huge importance and impact the discovery of IPC has made in the field of cardiovascular research over the last 30 years.
